# Mathieu Jaboulay's (1860–1913) contribution to xenotransplantation

**DOI:** 10.1111/xen.12765

**Published:** 2022-06-13

**Authors:** Daniel Rodger, Daniel J. Hurst

**Affiliations:** ^1^ Institute of Health and Social Care London South Bank University London UK; ^2^ Rowan University School of Osteopathic Medicine Stratford New Jersey USA

**Keywords:** kidney, Mathieu Jaboulay, pig, surgery, transplant, xenotransplantation

## Abstract

Mathieu Jaboulay (1860‐1913) was a professor of clinical surgery in Lyon, France who is best known for his development of vascular anastomosis and for conducting the first reported renal xenotransplantation experiments on humans, using pig and goat kidneys to treat end‐stage renal failure in 1906. His insights and pioneering techniques contributed significantly to allotransplantation and contemporary attempts at xenotransplantation. He is also credited with inventing several surgical instruments and novel surgical techniques that continue to influence vascular, general, and urological surgery to this day. However, this article will focus specifically on his notable contributions to xenotransplantation research and surgery.

## 1 INTRODUCTION

The modern and routine practice of solid organ transplantation is undertaken worldwide and is now a safe and effective means of treating acutely and chronically ill patients. In 2020, 129 681 organs were transplanted worldwide,^1^ representing many thousands of lives saved, but despite this, there remains a global shortage of organs to transplant. Therefore, inevitably, each year several thousand patients become too ill to receive a transplant and are removed from the organ transplant list or die while waiting. The lack of suitable donor organs has presented a problem ever since kidney transplantation first became a reality in 1954, when Dr Joseph Murray performed a kidney transplant between identical twins in Boston, Massachusetts.^2^

One promising alternative to allotransplantation—and a means of addressing the organ shortage problem—is xenotransplantation. Xenotransplantation is the cross‐species transplantation of organs, tissues, or cells between two different species.^a^ Notions of combining animal and human body parts are not new; it has ancient roots and can be found in the Hindu religion, Egyptian, Mesopotamian, Babylonian, Greek, African, Norse, and Roman mythology and folklore in the form of human‐animal hybrids, or humans with animal parts such as the minotaur and gorgon.^3^, ^4^, ^5^ Historically, experiments in xenotransplantation have been driven by clinical necessity in terminally ill patients in a final attempt to prolong life. In 1906, the first recorded solid organ xenotransplantation procedure in humans was performed by the French surgeon Mathieu Jaboulay. This pioneering surgery utilized his clinical experience and experimentation with novel techniques of vascular anastomosis, establishing him as a pioneer for modern xenotransplantation research and surgery today.

## 2 EARLY LIFE AND MEDICAL EDUCATION

**FIGURE 1:**
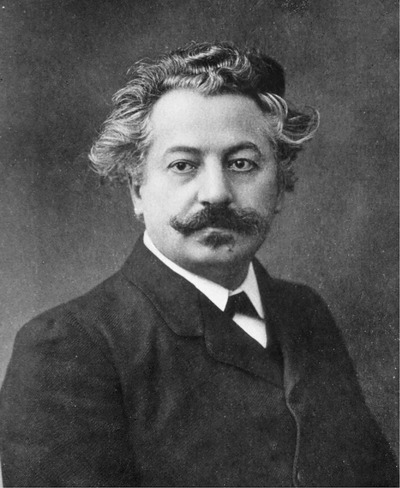
Mathieu Jaboulay (1860‐1913)

Jaboulay (Figure 1) was born on July 5th,^b^ 1860, in Saint Genis Laval near Lyon, France. Saint Genis Laval is famously named after Saint Genesius, the patron Saint of comedians, actors, dancers, and musicians, who had been tortured and beheaded during the reign of the Roman Emperor Diocletian (284‐305 AD). Jaboulay studied medicine at the University of Lyon Medical School, beginning his studies in 1880, passing his final medical examinations in 1884, and receiving his doctoral degree in 1886. That same year he was named Head of the Anatomy Department and two years later in 1888 he was awarded the prestigious title of Professeur Agrégé. During the 1890s, Jaboulay experimented with techniques on vascular anastomosis, co‐authoring a study with Eugène Briau (1870‐1951) in 1896 on the end‐to‐end suturing of blood vessels using a donkey carotid artery.^6^ Their technique involved an interrupted everting mattress suture (U‐stitch) incorporating all the layers of the blood vessel. He continued to practice surgery at the Hôtel‐Dieu de Lyon—a prominent hospital that dates back to the 12th century—where he became the Chair of Surgery in 1902, succeeding Louis‐Léopold‐Xavier‐Édouard Ollier (1830‐1900).^7^ ^.^ ^8^

Jaboulay was a popular lecturer and surgeon who was revered by his students and internationally among his peers, primarily for his originality and pioneering surgical techniques.^7^ Two of his most well‐known and influential students were René Leriche (1879‐1955) and the Nobel Laureate Alexis Carrel (1873‐1944), the latter of whom went on to develop and improve his work on vascular anastomosis. He therefore made significant direct and indirect contributions to vascular surgery through both his own work and that of his students.^9^

## 3 THE FIRST SOLID ORGAN HUMAN XENOTRANSPLANT

In 1902, Emerich Ullmann (1861‐1937) performed the first “successful” kidney transplant, when he removed and autotransplanted a dog's kidney to the vessels of its neck where it produced urine for a short period.^10^ Later that year, he also conducted dog‐to‐dog allotransplants as well as a dog‐to‐goat kidney xenotransplant that both produced urine. Ullmann used Payr's method, whereby the blood vessels are connected by tubes of absorbable magnesium metal. In the same year, the Vienna physician Alfred von Decastello (1872–1960) also performed a dog‐to‐dog kidney transplant. Ullman, like Decastello, would later abandon his work on transplantation.^11^ Decastello became famous for his co‐discovery with Adriano Sturli of the AB blood group. Building on this experimental work, Jaboulay would perform the first reported solid organ xenotransplants in humans.

In 1906, Jaboulay conducted the first reported clinical kidney xenotransplant that resulted in the production of urine. He believed that the use of animal organs could establish urine production as a means to help treat individuals with renal failure. The first transplant took place on the 24th of January in a 48‐year‐old woman whose clinical features included—oliguria, hypertension, headache, and hearing and vision loss.^12^ A pig was chosen as the source of the organ, and was killed three hours before the operation; followed by excision of the left kidney it was placed immediately in a warm artificial serum–what it consisted of is unclear.

Following dissection of the vessels in the fold of the patient's elbow, the pig kidney was grafted into the left antecubital space and left uncovered. The renal artery was connected to the central end of the humeral artery,^c^ and the renal vein to the central end of the median cephalic vein. Jaboulay believed that if kidney xenografts became part of established clinical practice, the elbow fold would serve as the ideal site due to the ease of access for surgery. Following the release of the Esmarch bandage, arterial blood flow was observed through the humeral artery into the renal artery. Upon the closure and dressing of the operative site, the kidney went on to produce 1500 mL of urine.^d^ Some secondary sources going back to the 1970s^e^ report that the xenograft only functioned for 1 hour, but, there is no evidence for this in any of Jaboulay's records.

On the third day, Jaboulay observed that the kidney was no longer functioning; it was removed on the same day. Following examination, Jaboulay identified the cause of the failure to be vascular thrombosis and erroneously lamented that the cause was his suturing technique.^12^ It would be decades before organ rejection would be definitively recognized as an immunological phenomenon,^13^, ^14^ which was the most likely cause of the xenograft failure.

The second of Jaboulays’ kidney xenotransplants took place nearly 3 months later on the 9th of April 1906. Although Jaboulay gives no definitive reason for changing his animal of choice from a pig to a goat, he does note that the goat kidney was smaller and had better quality vessels.^12^ In this case, a 50‐year‐old woman suffering from renal failure had a left‐sided goat kidney grafted to her left elbow fold.^f^ Despite an uneventful operation, the same outcome was observed, and the goat kidney was similarly removed on the third day. In both cases, the surgical wounds were left to heal by secondary intention.^g^

## 4 DEATH

Jaboulay—like Ullman and Decastello—would later abandon his work on xenotransplantation to continue his work on cancer, which was his primary focus in the latter years of his life.^15^ He unfortunately suffered an untimely death at the age of 53 on the 4th of November 1913 following a horrific train crash in Melun, France. The severity of the crash was such that his remains were not discovered until four days later on the 8th of November.^7^ In honor of Jaboulay's many accomplishments there is a road in Lyon named after him—Rue Jaboulay.

## 5 JABOULAY'S XENOTRANSPLANTATION LEGACY

The kidneys have historically been one of the primary organs of interest for transplantation. Because of their great need—the kidney is consistently the most transplanted organ in many countries. The immediate production of urine may indicate a successful transplant operation. They also have the surgical benefit of being vascularized by a single main vessel, the renal artery. It is perhaps easy and intuitive to deem Jaboulays’ two kidney xenotransplants to have been prima facie unsuccessful since neither resulted in long‐term function and patient survival. Nevertheless, arguably, they were in some meaningful sense “successful,” since in both cases the xenografts were vascularized and produced urine. Some of the human kidney transplants performed over the next few decades never produced any urine.^11^

After many failed xenotransplantation experiments using primarily non‐human primates as the source of organs. Jaboulay's early use of a pig for xenotransplantation would be re‐explored. Non‐human primates were a rational choice due to their genetic similarities to humans. However, the heightened risk of zoonotic disease transmission in primates, difficulty of large‐scale breeding, and greater hesitancy among the public for the use of primates in experimentation, in part led researchers away from non‐human primates. Surely a primary reason why Jaboulay used a pig in his initial experiment was because of its ready availability and few people would object to his killing a pig for this purpose. Nonetheless, his choice of pigs as the source of kidneys for clinical transplantation was taken up by others many years later.^21^ Today, the gene edited pig is the primary potential source of organs for clinical xenotransplantation.^h^ One observation made by Jaboulay was a particularly astute one. In his 1906 report, he commented that xenografts “…must create favorable conditions for blood clotting that the autograft avoids.” This correlates with studies carried out almost a century later in which significant discrepancies between the coagulation systems between pigs and primates were defined. These have only been overcome by the transgenic introduction of human coagulation‐regulatory proteins into the pig.^22^

Despite the complications and failures that plagued attempts at xenotransplantation for decades, Jaboulay had initiated the exploration of the pig—or goat—as a source of organs for humans. It has required solutions to numerous technological, immunological, and ethical hurdles, which over 100 years later, are now beginning to show the potential that many early proponents foresaw. Jaboulay, like many other pioneers before him, was willing to try something unconventional and risky in the hope of success and the restoration of health.

## ENDNOTES

a The original term used to describe this was heterotransplantation.

b There is some disagreement between the available secondary sources to whether Jaboulay was born on the 3rd or 5th of July, 1860.

c Jaboulay clearly recorded that he connected the renal artery to the patient's humeral artery: ‘*L'artère rénale fut soudée au bout central de l'artère humérale, la veine rénale au bout central de la veine médiane céphalique…*. However, this does raise some interesting questions, because strictly speaking, the humeral artery does not supply blood to the elbow.

d Some secondary sources state that the transplanted kidney did not produce any urine or that there was no visible function. However, Jaboulay in his 1906 article states that—a strong diuresis was the only result of this graft [‘*Une forte diurèse fut l'unique résultat de cette greffe…*’] and so it is clear that the first xenograft *did* produce urine.

e Examples of this claim can be found in the following sources.^16^, ^17^, ^18^, ^19^

f McCauley^20^ states that the goat kidney was grafted to the patient's thigh but Jaboulay describes it being grafted to the left elbow—‘*Je lui avais greffé au pli du coude gauche un rein gauche de chèvre enlevé…*’.

g It is not unusual to read that the two female patients died shortly after the surgery, but this is not described in Jaboulay's description of the two cases. He gives no indication that the patients were followed until the wounds healed, which by secondary intention would be around six weeks, though it is unlikely that they lived that long.

h For a recent example see Porrett et al.^23^
